# Semantic closure demonstrated by the evolution of a universal constructor architecture in an artificial chemistry

**DOI:** 10.1098/rsif.2016.1033

**Published:** 2017-05-17

**Authors:** Edward B. Clark, Simon J. Hickinbotham, Susan Stepney

**Affiliations:** 1 Department of Electronic Engineering, University of York, York, UK; 2 Department of Computer Science, University of York, York, UK

**Keywords:** artificial chemistry, universal constructor architecture, semantic closure, self-replicating automata, replicator–parasite systems, artificial life

## Abstract

We present a novel stringmol-based artificial chemistry system modelled on the universal constructor architecture (UCA) first explored by von Neumann. In a UCA, machines interact with an abstract description of themselves to replicate by copying the abstract description and constructing the machines that the abstract description encodes. DNA-based replication follows this architecture, with DNA being the abstract description, the polymerase being the copier, and the ribosome being the principal machine in expressing what is encoded on the DNA. This architecture is *semantically closed* as the machine that defines what the abstract description means is itself encoded on that abstract description. We present a series of experiments with the stringmol UCA that show the evolution of the *meaning* of genomic material, allowing the concept of semantic closure and transitions between semantically closed states to be elucidated in the light of concrete examples. We present results where, for the first time in an *in silico* system, simultaneous evolution of the genomic material, copier and constructor of a UCA, giving rise to viable offspring.

## 1. Introduction

The term semantic closure, introduced by Pattee [1], refers to the concept that a system can enclose its meaning within itself. Consider a string of DNA, with a given reading frame and start location we can say that the DNA, through its messenger RNA (mRNA), codes for a particular protein. This assumes particular triplets of DNA bases code for given amino acids. In biology, this encoding can and has evolved, altering the meaning of DNA by ‘rewiring the keyboard’ of the genetic code [2].

The key players behind semantic closure in biology are: the ribosome, transfer RNA (tRNA), DNA and mRNA. Each tRNA has three RNA bases that make up the anticodon, and is charged with one of the 20 types of amino acids. The ribosome acts on the mRNA, mediating numerous sequential tRNA interactions. The ribosome helps match the anticodon of the tRNA to the exposed bases on the mRNA, and appends the tRNA's payload of amino acid to the protein that is being produced [3]. The tRNAs, with the processes involved in expression, literally define the meaning of the DNA, through the mapping of three bases of DNA to one amino acid. There is a canonical mapping, commonly referred to as the codon table or ‘standard genetic code’ [4]. To change the mapping defined by the tRNAs is to ‘rewire the keyboard’ of the genetic code, and alter the meaning of the DNA. For a comprehensive review of the alternate codon tables, the organisms in which they occur, and the evolutionary forces that have been proposed causes of these codon changes in nature, see Knight [2].

All the mechanisms mentioned above (along with other relevant mechanisms such as post-transcriptional modification and RNA editing [5]) are carried out by molecules or complexes that are themselves encoded on the DNA, and make use of proteins translated in this way to provide or enhance their activity. This is the closed loop of meaning, as it exists in biology.

Synthetic biologists have been exploiting the power of the tRNA-based mapping by introducing synthetic tRNAs [4]. Making a change to the codon table might be expected to be deleterious to the host, and indeed the majority of *random* alterations would be deleterious [2]. Yet, there are 64 possible DNA triplets combinations and only 20 commonly occurring amino acids, and a ‘stop’. So each amino acid is mapped to a codon multiple times. It is possible to reassign one of these degenerate mappings to a synthetic tRNA with a novel payload without disrupting the composition of the molecules that are essential to translation and other vital processes [4]. Semantic closure, as a concept, is important for considering: how meaning was initially established for the translation of proteins; how it has been altered by evolution; the extent to which it can be exploited by synthetic biologists; and in particular for how to design viable synthetic cells from ‘the bottom up’, as such cells will include a de novo semantically closed system of molecules.

The genome, and consequently the mechanisms that define what the genome means, are subject to mutation and natural selection, allowing the potential for the meaning of the genome to be altered. Whenever biology has rewired the keyboard it has necessarily moved from one semantically closed state to another semantically closed state. Biology has demonstrated semantic closure by the *process* of moving between two semantically closed states. Having the architecture of semantic closure is *necessary* for this process, but it is unclear if it is *sufficient*.

Starting in the late 1940s von Neumann, in his work on self-replicating automata (SRA) [6], was the first to devise an artificial system that has the architecture of semantic closure. von Neumann's design has a constructor *A* that can interpret a fixed format genome *Φ*(*X*) and construct whatever the genome encodes, denoted *X*. von Neumann's design further includes: a copier *B* that can copy the genome; a controller *C* that controls the order in which the other machines operates; and an arbitrary payload *D* (so *X* = *A* + *B* + *C* + *D*). von Neumann speculated that mutation in the encoding of the constructor, copier or control would result in ‘sterile’ offspring [6, p. 86]. Hence it is not possible for that implementation to demonstrate semantic closure by transitioning between two semantically closed states thereby causing the meaning of the genome to be altered.

The design problems that faced von Neumann remain just as important to the field of artificial life as they did when he first considered them [7]. Since von Neumann's seminal work, there have been several further artificial systems that have successfully replicated with a constructor and a copier; we call this the universal constructor architecture (UCA). These systems can be categorized into two classes. The first [8] follows and refines von Neumann's original cellular automaton (CA) approach. More recently, an alternative approach uses automata chemistries (ACs) [9] to implement the UCA, examples of which we discuss below. While the UCA can be implemented in many ways, an implementation that is robust to mutation in either the CA or the AC paradigms has yet to be developed.

Baugh & McMullin [10] have implemented a UCA in Tierra [11]. Their implementation is capable of maintaining a population of UCA-based individuals in the absence of mutation. In their initial work, their UCA system collapses to the commonly observed Tierra self-copier behaviour whenever the mutation rate is higher than zero. The authors conclude that Tierra is not a sufficiently robust system under mutation to support the survival of a UCA, due to the emergence of abundant *pathological constructors* that replicate rapidly and dominate the ecosystem [10].

In Baugh's subsequent work [12], the physics of the Tierra system is altered by limiting reproduction to offspring that are the same length as the parent. The emergence of pathological constructors and self-copiers that were prevalent in the previous work has effectively been banned. The size of the lookup table used is increased to add redundancy, by increasing the number of nops (no operations) from two to 38, with no redundancy added for actual instructions. Under these restrictive conditions, mutations in the redundant nops section of the lookup table resulting in stable replication are reported. This is an interesting case as, in the absence of redundancy in the lookup table, mutations cannot give rise to a stable self-reproducer [12, p. 97]. No instances of mutations that alter the employed portion of the lookup table, leading to stable self reproducers, are reported. While it is undoubtedly a success in terms of altering the genotype–phenotype mapping, the success is limited to the non-employed areas of that mapping.

Hasegawa & McMullin [13] produced a similar implementation in Avida [14]. Again the UCA is observed to be viable in the absence of mutation. The authors conclude from their initial observations that their hand-designed seed has not been displaced by any mutation that preserves the UCA, but mutations that give rise to self-copiers are common [13,15].

While both the Avida- and Tierra-based implementations of the UCA successfully implement the architecture of semantic closure, the designs are brittle, albeit in a different way from von Neumann's: the offspring are not ‘sterile’, they merely abandon the UCA for the simpler strategy of reproduction by self-inspection.

Williams has developed a UCA within a new Haskell-based artificial chemistry (AChem) [16]. This is the first AChem explicitly designed to support the UCA. Although the system has the architecture for semantic closure, it does not currently have mutation. We conjecture that under mutation this chemistry will not demonstrate transitions between semantically closed states because the genotype–phenotype mapping is *directly encoded*: the genotype is simply the phenotype ‘in quotes’ with the minus form being the passive genotype (‘[]−’), and expression is the process of changing the minus to a plus (‘[]+’) to get the active phenotype of whatever is in the brackets, leaving no possibility of an alternative interpretation of the genotype.

In the work presented here, we describe a novel implementation of a UCA in the stringmol AChem [17]. The stringmol UCA reproduces itself even in the presence of mutation. In addition, we observe evolution of the stringmol UCA and numerous demonstrations of semantic closure, involving transitions between semantically closed states.

## 2. Definitions

Before we commence the description of our implementation of the UCA in stringmol, we must first clarify some terminology and give a description of the components of the UCA and their function.

### 2.1. Naming conventions

Semantic closure is a concept that can be applied in many disciplines, but the terminology to refer to important components of a semantically closed system is not consistent across disciplines. Table 1 shows how the components of the UCA are named in different domains. von Neumann has four classes of machine, plus a separate type of entity *Φ*, which acts as as the *description* of the other machines. In our stringmol UCA, we make no distinction between these types of *entities*. For clarity, we have included the analogous terms from biology in table 1, although our implementation is not a simulation of the biological behaviour.

**Table 1.** Comparison of naming conventions for some systems with semantic closure. An asterisk (*) denotes that control of the copying and expressing of genomic material is not localized in a single entity, but is instead distributed.

von Neumann SRA	stringmol UCA	biology
*A*: constructor	E: expressor	ribosome and tRNA
*B*: copier	C: copier	polymerase
*C*: control	*	*
*D*: ancillary	P: payload	proteome
*Φ* (*A*, *B*, *C*, *D*)	G: genome	genome

von Neumann's architecture includes a ‘Control’ machine, which coordinates the behaviour of all the other machines. There is no analogue of this machine in biology, because the behaviour of the ensemble is an emergent property of their local interactions. We follow biology here, and have no controller machine in our stringmol UCA. As in biology, control in stringmol is distributed across all the strings, with string-to-string interactions determined by binding sites on each of the strings.

Implementations of the UCA components vary widely in the literature. Usually [12,15,18], there is a single machine that contains connected ‘sub-assemblies’ of Expressor, Copier and Genome. We are aware of no implementations that explicitly include instances of any ancillary machine classes. It is important for the architecture to support at least one class of ancillary machines, though, in order that the system can interact with, and survive in, the world.

### 2.2. The universal constructor architecture

Our stringmol UCA has four classes of machine (see [19] for more details on what we mean by machine). We use the following terminology to describe them:

— C, *Copier*: a machine that can interact with a Genome machine and create a copy of it. The biological analogy is a polymerase.
— E, *Expressor*: a machine that can read the specification of any machine *X* encoded on the Genome, and construct an instance of *X* from that specification. Note that the form of a machine and the form of its encoded specification are distinct. The biological analogy is the ribosome and tRNA assemblage expressing a protein.
— G, *Genome*: a machine that holds the description of all the other machines in the system. (The Genome is its own description; in this sense it is a different ‘type’ from the other machines. However the Genome and the other machines all exist as strings in the stringmol implementation.)
— P, *Payload*: any ancillary machines that are not involved in the reproduction of the system, but are reproduced by the system.

All machines co-define each other's function when bound in a particular way, and the behaviour of a machine can change as the system evolves. The UCA replicates in two stages: the Copier machine copies the Genome; the Expressor machine interprets this new copy, and constructs all the machines it encodes.

## 3. The stringmol implementation of the universal constructor architecture

Having defined the UCA in the previous section, we now describe our approach to realizing it in stringmol. We begin with an overview of the stringmol automata chemistry (for a complete description see [20], or for an online tutorial visit http://stringmol.york.ac.uk/), then present the components of the stringmol UCA system.

### 3.1. The stringmol automata chemistry

Stringmol [20] is an automata chemistry [9] in which the ‘molecules’ are programs encoded as strings of *opcodes* (single characters, each of which specifies a computational operation to be performed). A stringmol chemistry operates in an abstract container, in which multiple pairs of molecular strings interact with each other. An aspatial physics engine gives pairs of molecules the opportunity to *bind* using a ‘soft’ matching algorithm, where less precise matches have a lower but non-zero probability of binding. On binding, the initial state of four pointers per string (Instruction, Flow, Read, Write) that control program execution are set. After binding, the molecular program executes, using both strings as determined by the sequence of opcodes. There are 33 opcodes in the stringmol language. Seven opcodes are functional: ‘?’, ‘$’, ‘^∧^’, ‘%’ ‘}’, ‘>’ and ‘=’, used for executing programs. These opcodes manipulate the pointers. There are 26 nop opcodes, A–Z, which do nothing when the instruction pointer executes them, but are used for binding sites and modifiers of the functional opcodes.

An energy flux places an effective carrying capacity on the number of molecules in the system (although this depends on the size and properties of the molecules). Each opcode consumes a unit of energy when executed.

A stochastic decay function removes molecules from the system with a fixed probability. Species of molecules must somehow be reproduced or they will disappear from the container.

It is possible for the container to ‘die’ when a mutation destroys a self-maintaining cohort of molecules and no molecules remain in the container.

Mutation in stringmol happens when a molecular program uses the copy opcode ‘=’. A small chance of error is built into this operator, with the effect that a symbol *X* at the read pointer is mis-copied, and a different symbol *Y* is written to the location indicated by the write pointer.

For full details of the way stringmol has been used to explore replicase systems, see [17]. We now describe the stringmol UCA.

### 3.2. The universal constructor architecture in stringmol

In the stringmol UCA, each component of the UCA (Expressor, Copier, Payload, Genome) is implemented as a molecular species. The container is populated with multiple instances of each species, which interact to replace molecules that are destroyed.

**Figure 1.**
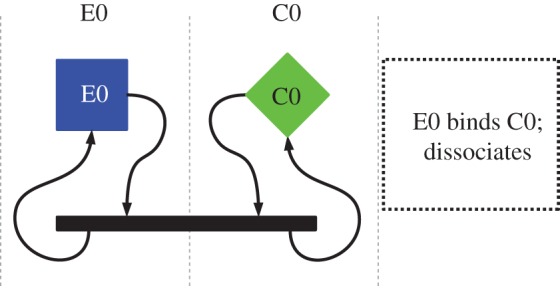
Null reactions in stringmol UCA. Boxes represent a single molecule. Black bars represent a reacting pair of molecules. Black arrows show change of state. Each shape represents a single molecule. Each molecular species has its own column and its own colour. Copiers (C0) are shown as diamonds. Expressors (E0) are shown as squares. Two molecules bind into a reaction state. No new molecules are produced in this case, and the original reactants dissociate back to their original state.

There are 16 possible pairwise interactions, most of which have been designed to terminate quickly with no product, as illustrated in figure 1, where the copier and expressor molecules react and dissociate with no product and no change to the reactant molecules. The stringmol UCA requires both copying and executing the program on the genome, and the stringmol instruction set allows strings to be interpreted as data or executed as program.

There are only two reactions in this initial system that create new molecules: copier–genome, which results in a new genome; and expressor–genome, which results in a new copier, expressor and payload.

**Figure 2.**
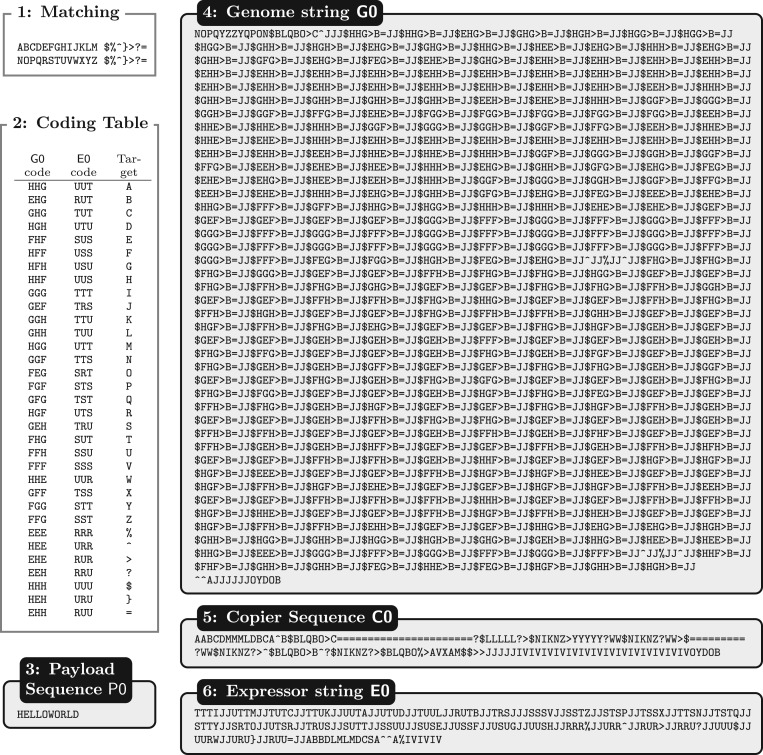
Stringmol UCA design. *Box 1: Matching* is used to determine pairs of binding sequences. Opcodes in the top row are an exact match to the corresponding entry in the bottom row. *Box 2: Coding Table* for genome: column 1 is the genome coding, column 2 is the complementary string on the expressor, column 3 is the opcode that the triplets code for. *Boxes 3–6* show the sequences for the Payload, Genome, Copier and Expressor molecules.

The four molecules that are part of the design are shown in figure 2, boxes 3–6.

#### 3.2.1. The genome molecule

The sequence of the genome string is shown in figure 2, box 4. The first 23 and last 14 opcodes form the bind site and functional opcodes to arrange the pointers for gene expression.

The main body of the genome (3656 opcodes) is devoted to encoding the other machines. The encoding efficiency is nine to one: it takes nine characters on the genome to encode one character on the expressed machine. For example, the sequence JJ$GEH>B= is a coding block for the ‘S’ opcode. There are four components to the encoding:

(1) JJ: protects against unwanted bindings in the coding region.
(2) $GEH: specifies a search in a lookup table encoded on the expressor molecule. The $ is the search opcode, and the GEH is the modifier that specifies the search. The Flow pointer is moved to the best match on the expressor molecule.
(3) >B: moves the Read pointer to the Flow pointer on the expressor molecule.
(4) =: copies the opcode at the Read pointer to the position of the Write pointer. This appends the new opcode S to the end of the new string that is being expressed.

A genome molecule can encode more than one string. This is achieved via a JJ^∧^JJ%JJ^∧^ substring, which cleaves the newly expressed string from the end of the genome and then resets the pointers for continued expression of subsequent strings.

#### 3.2.2. The copier molecule

**Figure 3.**
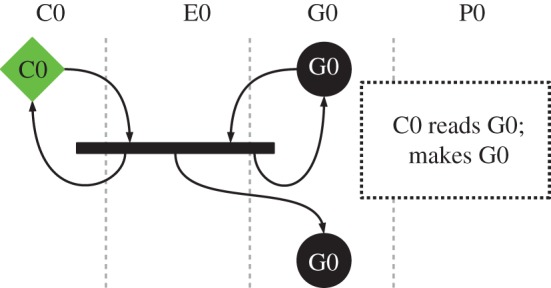
Normal operation of the copier machine in stringmol UCA. Key as in figure 4. Here, the copier machine C0 creates a copy of genome G0.

The copier can interact with all other molecules in the system, but it is designed to copy only the genome, as shown in figure 3. When the copier interacts with the genome, the binding function causes the pointers to be arranged in such a way that the genome is copied. When the copier interacts with another copier, or an expressor or payload molecule, the reaction instead terminates quickly with no product and no changes to either of the reactant molecules. The expressor–copier interaction is shown in figure 1. These null interactions are a result of binding using the sequence IVIVIVIVIVIVIV at the end of the molecule. Figure 2 box 1 shows that nop I binds to V, so this sequence binds strongly to the same sequence on another molecule.

#### 3.2.3. The payload molecule

The payload molecule is included in the design, mirroring the work by von Neumann. The payload is symbolic of any molecule, such as those involved in discovering and utilizing resources for the continued function of the system. As proof of principle, we chose HELLOWORLD as the payload for these experiments, as is traditional in computer science. Unlike the copier and expressor, it does not have a bind sequence IVIVIVIVI on it. Where HELLOWORLD interacts with other strings, its reaction is null.

#### 3.2.4. The expressor molecule

**Figure 4.**
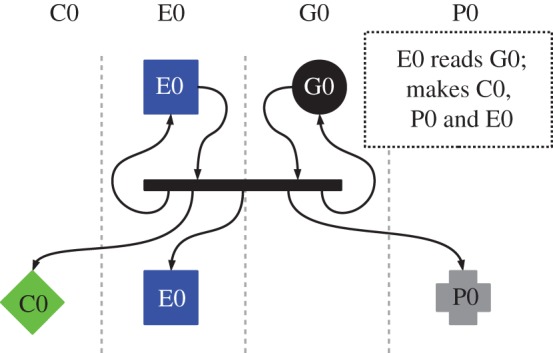
Normal operation of the expressor machine in stringmol UCA. Each shape represents a single molecule. Each molecular species has its own column and its own colour. Copiers (C0) are shown as diamonds. Expressors (E0) are shown as squares. Genomes (G0) are shown as circles. Payloads (P0) are shown as crosses. Black bars represent a reacting pair of molecules. New molecules produced during a reaction are shown below the bar. Arrows show change of state. The Expressor binds to the Template machine, called ‘Genome’ in the figure. The Genome contains descriptions of the copier, the expressor, and payload. The expressor reads these instructions and creates new instances of these machines.

The expressor molecule is responsible for constructing new instances of all the UCA molecules, except the genome, including the expressor molecule itself. The reaction between the genome and expressor is shown in figure 4.

The sequence of the expressor is shown in figure 2 box 6. The first 231 codes in the molecule specify the lookup table that translates the three-letter codon to the opcode it represents. These are analogous to tRNA in biology. Each entry in the lookup table consists of six opcodes. For example, in the sequence TRUSJJ:

(1) TRU is an exact match to GEH, which means that when the search is executed with the Flow pointer on the expressor, the Flow pointer is moved to the character immediately after TRU.
(2) S is the target opcode to be appended to the molecule currently being expressed.
(3) JJ protects against unwanted bindings in the lookup table.

After the lookup table on the expressor, there are three further regions, governing the binding to the genome (ABBDLMLMDCSA), the code for cleaving the final molecule encoded at the end of the genome (^∧^^∧^A%), and the code for handling binding to copier and expressor molecules in a safe manner (IVIVIV). These sections of the string are somewhat analogous to the ribosome in biology, since they deal with initiating and completing expression.

Formulating the expressor molecule in this way allows the genome to code for an arbitrary number of machines that are expressed sequentially in each reaction between a genome string and expressor. The genome shown in figure 2 box 4, when in a reaction with the expressor (figure 2 box 6), encodes three machines: the copier (figure 2 box 5), the expressor, and the payload (figure 2 box 3).

## 4. Method

The version of the stringmol simulation engine used here is the same as in previous publications [17,20] with three necessary changes: (i) the per-copy mutation rate is reduced to 0.5 × 10^−6^; (ii) the molecular decay rate is reduced to 0.24 × 10^−4^; (iii) the internal limit on maximum string length (previously 2000, shorter than the seed genotype) is increased to 200 000, increasing the memory footprint of simulations. All three changes are necessary to accommodate the approximately 100-fold increase in the number of characters in the UCA seed system compared with the previously published replicator seed which had 65 characters [17]. The decay rate has to be altered to increase the probability that molecular programs complete before the strings involved decay. The mutation rate has to be reduced to increase the probability of an unaltered offspring.

Each container run was initialized with 150 unbound strings: 50 each of Copier C0, Expressor E0, and Genome G0 (as defined in figure 2). The Payload P0 had an initial population of 0. Each container was allowed to run until the population of the container fell to zero.

In total, 500 containers were run. Plots of the populations of each run were produced and manually inspected for instances where one or more of the strings of the running UCA had been fully displaced, while maintaining a viable container. For each instance of displacement, the lineage of the strings of the new system were traced way back to the seed UCA, the origin of the mutation was noted, and the mechanisms leading the generation of this successful new string were analysed.

## 5. Results

The 500 runs produced 39 examples of container-wide takeovers, where one or more seed species is replaced by a mutated version while maintaining a viable UCA. Further breakdown of these results is given in table 2. Thirty-two of the takeovers involved mutations in either the copier, expressor or both. Mutations in the copier or expressor that lead to viable UCAs are of particular significance as von Neumann had speculated that such mutations would exclusively lead to non-viable systems [6]. Whether von Neumann was making a general statement or a specific statement, it has largely held true until now.

**Table 2.** Frequency of semantic takeover phenomena in 500 runs of stringmol UCA. Four of these cases are discussed in detail in the sections noted.

	machines changed						
initial mutation while …	C0	E0	G0	P0	frequency	section	row label
… expressing (e.g. E0 + G0 → E1 or C1 or P1)		•			1	5.1	(i)
		•		•	2	—	(ii)
	•	•			1	—	(iii)
… copying (e.g. C0 + G0 → G1)		•		•	2	5.2	(iv)
		•			4	—	(v)
	•	•			6	5.3	(vi)
	•		•		6	—	(vii)
	•	•	•		4	5.4	(viii)
		•	•		6	—	(ix)
			•		7	—	(x)

**Figure 5.**
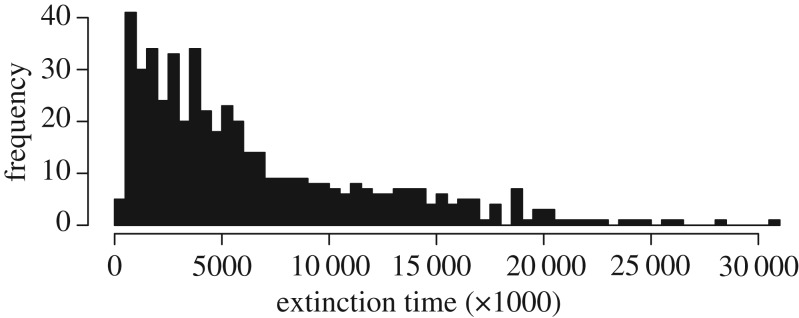
Time to extinction for 500 trials of stringmol UCA.

Figure 5 shows a histogram of extinction times for the 500 runs. The mean is 6 572 488 time steps, and the median is 4 665 000. The longest run lasted over 30 million time steps, which equates to approximately 1.5 billion instruction executions.

There were no examples of the payload string HELLOWORLD being evolved out of the genome. The payload string makes no positive contribution to either the copying of the genome string or the expression of the other strings from the genome string. Given the dispensable nature of the payload string one might have expected it to be evolved out. Owing to the design of the interaction between the expressor and the genome string (§3.2.4), individual strings that are to be expressed cannot be trivially bypassed by mutations to the system. The payload string can undergo neutral mutation, including heritable character deletions, which could eventually entirely alter or remove the payload string.

One might expect, given the recent work by McMullin's group [10,12] in comparable artificial chemistries to stringmol, that the UCA in stringmol would either collapse to a replicase or be overrun by obligate parasites. In the 500 runs, there were no examples where the UCA system was displaced by a replicase system. Were the two systems to compete directly in a single container, the replicase system would win because it is able to reproduce much more quickly. Therefore, the lack of observation of the system collapsing to a replicase indicates that no viable replicase systems evolved during the 500 runs. We can conclude that the design of seed UCA is sufficiently well disconnected from the attractor of replicase systems to make them not trivially reachable by mutation. We speculate that with a sufficiently long series of mutations it would be possible to traverse the landscape of viable systems and eventually make it possible to collapse a UCA to a replicase system, but this is not observed in our results due to the very small probability of such a series of mutations occurring during a single run.

The standard mode of death in previous studies conducted in the stringmol system [17,20–22] is ‘death by parasites’, which gives rise to a characteristic spike in the parasite population that is causally linked to the death of the container. Death by parasites did not occur in any of the 500 runs of the stringmol UCA. Instead, container death appears to occur as a result of an explosion in the diversity of the strings in the container followed by the decay of the viable system as the diverse set of strings compete. While this mode of death has not been seen in stringmol previously, it has been observed in other artificial chemistries [23]. The mode of death is referred to as ‘bureaucratic death’, as the system simply collapses under its own weight with no clear culpability.

Bureaucratic death in stringmol UCA stands in stark contrast to death by parasites in the earlier stringmol replicase systems, where there is an easily identifiable parasite string that exists in large numbers. The change in the mode of death in the stringmol AChem is significant, as it is generally thought that each AChem has characteristic behaviours that are inescapable. This result demonstrates that such behaviours are not universal attractors in the design space of possible seed systems. This is a cautionary tale, demonstrating that properties are sometimes incorrectly attributed to the underlying AChem, when they should actually be attributed to the *seed system* in that AChem.

Of the 39 evolutionary events shown in table 2, four are initiated by a mutation while expressing (rows (i)–(iii)). Thirty-five are initiated by a mutation while copying the genome (rows (iv)–(x)), giving rise to heritable mutations in a standard way: the error is replicated, and is then subject to selection. The mutated genome is retained 23 times in the 500 trials: rows (vii)–(x) of table 2.

The other 16 cases (rows (i)–(vi)) have acquired a heritable mutation that is not encoded on the genome string, because G0 is unchanged. One of these in particular stands out: row (i). The initial mutation that gives rise to the heritable change to the system happens during expression, and the only string in the system that is altered is the expressor. This is the cleanest possible example of demonstrating semantic closure. The genome originally encoded the seed expressor, now it encodes a different expressor, but the genome string itself is not altered at any time, *only the meaning of the genome string has been altered*. We present a detailed examination of four of the 39 takeovers shown in table 2, beginning with this quintessential case of semantic closure.

### 5.1. Expressor takeover

**Figure 6.**
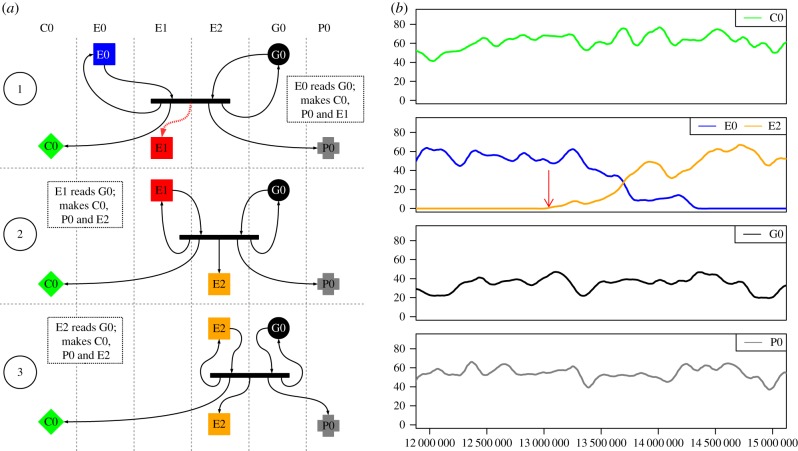
Semantic change without mutation of the genome. (*a*) *Reactions.* Key as in figure 4. (1) Mutation while expressing G0 leads to E1 instead of E0. The error is shown with a dashed red arrow. (2) E1 interprets G0 differently from E0. Where E0 reads G0 as coding for E0, E1 reads the gene as coding for E2. Thus E1 expresses E2 *without error*. (3) E2 interprets G0 as a specification for C0, P0 and E2. (*b*) *System dynamics.* Smoothed using Loess smoothing, and showing only the molecular species from the left panel. Timing of step 1 is shown with a red arrow. In this example, the genome (black), the copier machine (green) and payload (grey) all remain unchanged. The original expressor (blue) is replaced by a mis-expressed expressor (orange).

In run 121 (located in the data repository listed in §7, results folder 2.1a), the seed UCA C0, E0, G0, P0 was displaced by the system C0, E2, G0, P0. Figure 6*a* shows that a mutation (a copy error during expression, reaction 1, dashed arrow) results in the creation of a new expressor, E1. The new molecule, E1 is a fully functioning expressor, but as E1 encodes a different lookup table from E0, it translates G0 differently, as shown in step 2 of figure 6*a*: note that G0 is unchanged. When E0 interprets G0 it expresses C0, E0 and P0 (when error-free). However, when E1 interprets G0 it expresses C0, E2 and P0, as shown in step 3 of figure 6*a*. E1 is a transient species as it does not interpret G0 to produce more copies of E1. By contrast, when E2 interprets G0 it expresses C0, E2 and P0, as shown in step 3 of figure 6*a*, so E2 is not *necessarily* transient.

Both E0 and E2 are capable of increasing their population by interacting with G0. The expressors, having the same binding probability and expression efficiency, compete in an unbiased random walk, as shown in figure 6*b*. When the E0 population falls to zero and only E2 remains, the *de facto* situation is that the meaning of G0 has been altered during the run. The system has moved from one semantically closed state (C0, E0, G0, P0) to another semantically closed state (C0, E2, G0, P0), with different interpretations of what G0 encodes.

As *only* the expressor is altered in this example, it follows that the meaning of the G0 is altered in a somewhat superficial way. It is not unreasonable to consider this to be a change in a junk region as this alteration in the meaning of G0 is not represented in the other strings. This example is similar to the transitions observed by Baugh [12].

### 5.2. Expressor and payload takeover

**Figure 7.**
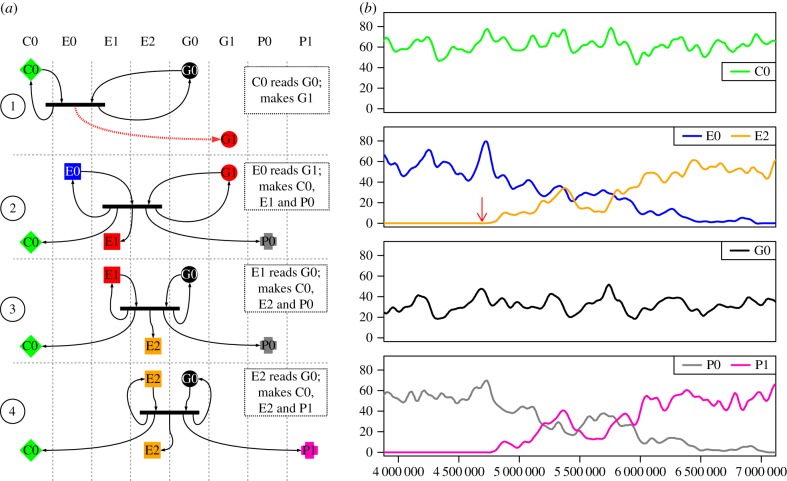
Semantic change via mutation on the genome. Key as in figures 3 and 6. (*a*) *Reactions.* (1) Mutation while copying a G0 creates G1. (2) E0 creates the machines specified on G1, one of which is the new machine E1. (3) E1 reads G0 as coding for E2. Thus E1 expresses E2 *without error*. Similarly, E1 reads the G0 for payload as P1. (4) E2 interprets G0 as a specification for C0, E2 and P1. (*b*) *System dynamics.* Timing of step 1 and 2 are shown by a red arrow. The original expressor E0 (blue) and payload P0 (grey) are replaced by E2 (orange) and P1 (pink), respectively. In this example, both the genome (black) and the copier machine (green) are unchanged.

In run 9 (located in the data repository listed in §7, results folder 2.1a), the seed UCA C0, E0, G0, P0 was displaced by the system C0, E2, G0, P1. This takeover is initiated by a mutation while copying G0 giving rise to G1 (figure 7*a*). G1 differs from G0 in the region that encodes the expressor. An interaction between this new genome G1 and the original expressor E0 creates a novel expressor E1. G1 fails to fix itself in the population. E1 interprets G0 differently from E0, giving rise to a further version of the expressor molecule, E2, and a new version of the payload molecule, P1.

The expressor E1 is necessarily transient as it cannot increase its own population. By contrast, E2 is able to increase its own population through interaction with G0. The dynamics of the takeover are shown in figure 7*b*. Note that neither G1 nor E1 are ever present in great numbers. The plot shows that the population of G0 and seed copier C0 remain stable throughout the takeover. The original expressor E0 and the expressor E2 engage in a random walk, which E0 loses. The prosperity of P1 is directly linked to E2 as E2 interprets G0 as encoding P1 instead of P0. As a consequence of E2 winning the random walk, P0 becomes extinct and is replaced by P1.

In this example, we see how alterations in the expressor can lead to novel interpretations of the genome giving rise to pleiotropic effects. Not only has the meaning of the genome been altered, but the consequences of the novel interpretation of G0 are not limited to the expressor molecule, but can impact other molecules that are encoded on G0. In the stringmol UCA, the payload molecule P0 serves no function, so it can be replaced easily, so long as the new molecule is not deleterious to the system.

### 5.3. Expressor and copier takeover

**Figure 8.**
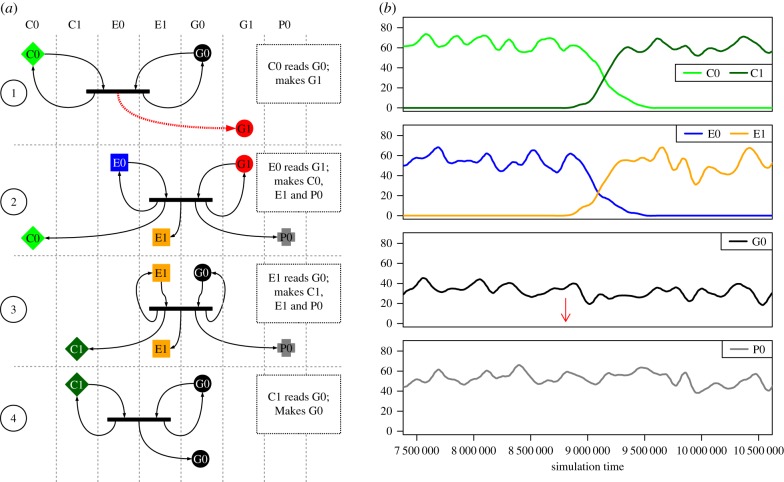
Semantic takeover of expressor and copier molecule. Key as in figures 3 and 6. (*a*) *Reactions*. (1) Mutation while expressing a G0 creates E1. (2) E0 creates the machines specified on G1, one of which is the new machine E1 *without error*. (3) E1 reads G0 as coding for P0, E1 and C1. Thus E1 expresses C1 *without error*. (4) C1 copies G0 *without error*. (*b*) *System dynamics*. Timing of step 1 is shown by a red arrow. The original expressor E0 (blue) and copier C0 (green) are replaced by E1 (orange) and C1 (dark green), respectively. Both the genome (black) and the payload (grey) are unchanged.

In run 106 (located in the data repository listed in §7, results folder 2.1a), the seed UCA C0, E0, G0, P0 was displaced by the UCA C1, E1, G0, P0. This takeover is initiated via a mutation while copying the genome G0 (figure 8*a*). This new genome G1 interacts with the expressor E0 and creates a new expressor E1. This new expressor molecule reads the original genome G0, but interprets it differently from the original expressor E0, giving rise to a new version of the copier molecule C1, as well as increasing the population of E1. Unlike the previous examples, E1 is not necessarily transient as it is able to increase its own population.

The dynamics of the takeover is shown in figure 8*b*. This shows that the population of the seed genome G0 and seed payload P0 remain stable throughout the takeover. The seed expressor and copier molecules E0 and C0 are driven to extinction, while the new versions of these molecules E1 and C1 complete the takeover.

Again we see an example where a novel expressor, E1, displaces the original expressor E0. However in this example, E1 is interpreting the genome G0 in such a way as to give rise to an altered, but functional, copier C1. This is new ground: it demonstrates not only a change in semantically closed states, but also that the stringmol UCA system is flexible enough to accommodate mutations in both the key machines for replication. The evolutionary potential of such a system is vast as both the meaning of genetic material and the mechanism of copying are subject to evolution.

### 5.4. Genome, copier and expressor takeover

**Figure 9.**
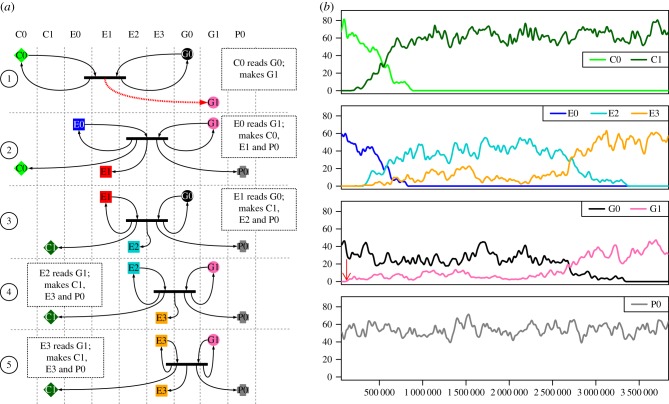
Semantic takeover using copier, genome and expressor. Key as in figures 3 and 6. (*a*) *Reactions*. (1) Mutation while copying G0 creates G1. (2) E0 creates the machines specified on G1 *without error*, one of which is the new machine E1. (3) E1 reads G0 as coding for P0, C1, E2. Thus, E1 expresses C1 and E2 *without error*. (4) E2 reads G1 as coding for P0, C1, E3. Thus, E2 expresses C1 and E3 *without error*. (5) E3 reads G1 as coding for P0, C1, E3. Thus, E3 expresses C1 and E3 *without error*. (*b*) *System dynamics*. Timing of step 1 and 2 are shown by a red arrow. The original expressor E0 (blue) is replaced by E2, which in turn is replaced by E3.

In run 87 (located in the data repository listed in §7, results folder 2.1d) the seed UCA C0, E0, G0, P0 is displaced by UCA C1, E3, G1, P0. The takeover differs from the previous example, taking place on a longer timescale, see figure 9*b*, and involves more steps on the critical path between the seed UCA and altered UCA. It also fixes a novel genome in the population, which we have not examined previously. This takeover is initiated when the seed copier C0 introduces a mutation while copying G0 to produce G1. Unlike our previous examples, G1 does eventually fix itself in the population, but only after a lengthy random walk with G0.

The fact that G0 and G1 exist together for an extended duration (figure 9*b*) may be important in determining why this example is more intricate than the previous examples. Any expressor will have the opportunity to interact with both G0 and G1, with potentially different outcomes.

When we examine the path from E0 to E3, the extended coexistence of G0 and G1 is indeed a factor: E0 translates G1 to make E1; E1 translates G0 to make E2 and C1; E2 translates G1 to make E3 and C1.

Looking at the dynamics of the takeover (figure 9*b*), we can make several observations. There is a visible lag between the appearance of G1 and E2. Early interactions between G1 and C0 will have helped to establish G1 in the population by increasing its numbers. In the stringmol UCA, it is not possible for a given genome string to be copied and expressed simultaneously, so while G1 is being copied it is not available to be expressed.

It is also possible to observe in the dynamics that the population of C0 is dependent on E0. When the population of E0 falls to zero, only E2 and E3 persist, but they both interpret G1 (and G0, interactions not shown) to encode C1 (figure 9*a*, steps 3 and 4). A further dependency of E2 on G0 can be observed in the dynamics.

Once all the dynamics of the takeover have concluded, a stable UCA system remains where E3 translates G1 to make E3, C1 and P0 (figure 9*a*, step 5) and C1 is able to copy G1 (interaction not shown). In this takeover we have observed mutation to all of the three strings that are *necessary and sufficient for replication* of a stringmol UCA system, while maintaining an unaltered payload string.

## 6. Discussion

We have presented a novel stringmol system modelled on the UCA first explored by von Neumann [6]. The stringmol UCA system has demonstrated the concept of semantic closure by evolving the *meaning* of its genomic material, transitioning from one semantically closed state to another. Analysis of our dataset has revealed the evolution of a seed UCA to another viable UCA system where all the *necessary and sufficient components for replication* have evolved simultaneously (§5.4).

The stringmol UCA has given rise to a mode of container death that has not previously been observed in stringmol, while completely overturning both death by parasites and collapse to a replicase system. In all previous work on the stringmol system, death by parasites has been observed as the cause of the system-wide death. In the stringmol UCA, we instead observe a mode of death characterized by an explosion of diversity, which we refer to as *bureaucratic death*, due to system collapsing without any obviously culpable string. The complete lack of parasites from the stringmol UCA merits further investigation.

Bureaucratic death starts with a self-replicating UCA with high fidelity expression. A single mutation, if this mutation alters the expression machinery itself, can potentially lead to a cascade of progressively mis-expressed expressors that go on to mis-express more expressor variants, and so on, until the system collapses. This would seem to be the opposite of Eigen's paradox [24], which is concerned with how high fidelity copying can arise from a system with low fidelity copying. The two phenomena may well be related, as they are mirrors of each other in terms of the two halves of replication: copying and expression.

Early life can be posed as the problem of a perplexing transition from a system that can replicate genomic material, but with only low fidelity, evolving to a system that can replicate genomic material with much higher fidelity [25]. Let us assume that this early life also has some process by which the genomic material is expressed. Instead of the one parameter problem, of the accuracy of replication of genomic material, there is now a two parameter problem, with the stability of semantic closure being the second parameter.

It is reasonable to speculate that early life would have had poor fidelity of expression of proteins, and potentially of RNA. The ‘double-sieve’ editing mechanisms ensure tRNAs are loaded with the incorrect amino acid as infrequently as once in 10^−4^–10^−5^ [3]. High fidelity expression is not something that should be taken for granted when considering early life, as it has been acquired through the evolution of numerous processes that have made incremental improvements over billions of years. For low fidelity or even non-specific loading of amino acids onto tRNAs, the genome that we think of as *defining* the phenotype of an organism would have been more of a guideline than a rule. As a consequence, low fidelity replication of a genomic material may well have had less impact on a proto-organism: many mutations would affect the phenotype only as far as altering the probabilities of what would be expressed. While the path through this two parameter space (fidelity of replication versus fidelity of expression) is not immediately apparent, it significantly recasts the problem of considering the emergence of life as we know it.

The stringmol UCA has proved to be more successful, in terms of demonstrating its evolutionary potential, than the previous attempts in Tierra, Avida or von Neumann's automata. It could be that the stringmol language is better suited to an evolvable UCA design; although the language was designed for a replicase system, it was amenable to being repurposed for the design of a UCA without any changes to the language, only to the seed. Both the Tierra and Avida UCA designs introduced new operators, altering the languages to more easily achieve the goal (or to make the goal possible at all, as the case may be).

Required changes to a language for a given task can be used to highlight design issues in that language. In §4, we detailed the necessary changes to stringmol mutation and decay rates, exposing the fact that these are not under the control of systems designed in stringmol. Revision of the stringmol language, or development of new languages for study in this area, should attempt to make both these features properties of the system that is designed using the language, rather than imposing them from outside.

It is difficult to prove what the *necessary* factor is in the stringmol language or the stringmol UCA design itself that is *sufficient* to give rise to transitions between semantically closed states. We can however speculate that it is the softness of the template matching algorithm in the stringmol language [21], in conjunction with a semantically closed architecture, that facilitates transitions between semantically closed states.

One aspect of the stringmol language that is almost certainly beneficial, especially for considering semantic closure, is that the concept of ‘absolute meaning’ is absent. Stringmol's pairwise reaction system implies that a molecule has a meaning only when bound to the molecule that confers that meaning upon it. We can call a string ‘an expressor’, for purposes of exposition, but it is only really an expressor in the context of a genome string. In the context of other strings it is not an expressor. In stringmol, two strings react and a single program is defined based on the strings and the initial pointer positions. However, each string can take part in many reactions and be a partner in defining many programs. Stringmol allows great complexity, as a container with *n* different species of strings defines *n*^2^ programs (some of which may be trivial). As these programs may be tried in any order, there is a minimum level of robustness that needs to be designed into any system. This is why stringmol does not have an explicit ‘control’ machine (table 1): control is inherently distributed across all the strings, nominally in the strings' binding sites. In this way stringmol is similar to biology: proteins have binding sites, but if sufficient non-binding site amino acids were altered, their function would be altered or degraded, or new binding sites for new partners would be created.

Continuing to consider meaning we can reflect on what is meant by a universal constructor, specifically, what does ‘universal’ mean in this context. In the stringmol UCA design, the expressor E0 can express any string that can be written in the stringmol language; its lookup table represents the full complement of stringmol's 33 opcodes. In this sense, it is a universal expressor. However, when we look in detail at the results of our experiments, we see that certain opcodes can become lost. The expressor can however express all the things that it can express; it is necessary to become self-referential here as the issue of self-defined universality is the point under consideration. One might consider the ribosome a universal constructor because it can construct all the things it can construct. However, there are many chemicals that it cannot make. We argue that the ribosome is not a universal constructor as it can only construct sequences of amino acids. It constructs everything that the DNA encodes, but it imposes the *meaning* of what the DNA is encoding.

Transitions in semantically closed states in biology as a result of alterations in tRNA are well documented [2]. These alterations are observable in the genetic record in the part of the DNA that corresponds to the tRNA. Based on the results we have presented here, it is possible in stringmol to generate changes in semantic closure that do *not* appear in the genetic record, but arise through inaccurate expression. Critically, our results show that, while many of these errors in expression are what we refer to as ‘necessarily transient’, some give rise to ‘covert’ *heritable changes in expression that are not reflected in the genome*. In biology, errors in expression are considered to be either deleterious or not heritable, as only information stored on the genome is considered heritable: these are considered to be always necessarily transient errors. We speculate that errors in expression that would lead to such covert changes in semantic closure in biology may well have occurred, but have gone unobserved, or are possibly even unobservable in the genetic record.

Based on the mechanisms by which these covert transitions occur in the stringmol UCA, we propose two mechanisms by which more subtle changes in semantic closure could be discovered to occur in biology, that is, changes that occur by some mechanism that does not include alteration of the DNA that encodes tRNA. For the purposes of seeding the imagination, we propose two ways in which this might occur: one route is via RNA and the other via proteins.

It is possible for RNA to be altered by RNA editing; it has been shown that tRNA is edited in this way [26]. It is possible that evolution in RNA editing mechanisms could alter the editing of a tRNA in such a way as to change the charging specificity (the amino acid that is attached to the tRNA could be altered) while the anticodon is unchanged. While in principle this is detectable through the genetic record, as the change to the RNA editing molecules would be observable in the genetic record, the codon table would be altered without any change to the DNA that directly encodes the tRNA, and so the importance of the change in RNA editing might easily be overlooked. Examination of the genetic record for RNA editing mechanisms and their tRNA interactions could provide direct evidence for a covert change in semantic closure having actually occurred in biology.

Now let us consider an example where a transition between semantically closed states could occur in biology with no evidence at all in the genetic record. We consider as a base for our example the streptomycin-dependent phenotypes of *E. coli* [27]. The presence of the antibiotic streptomycin modulates the structure of the ribosome, causing read through errors, altering the interpretation of the DNA. Investigation of this phenomenon of structural alterations of the ribosome led to the following postulate made in 1965: *‘It is postulated that some of these alterations provide the ability to misread specific codons.’* [27], which has subsequently been supported by clear evidence [3]. If we accept that the ribosome can be modulated by alterations to proteins so as to change the meaning of (‘misread’) specific codons, and if we also accept that chance errors in the expression of these proteins can occur, then it follows that the possibility exists that a chance error in expression of a protein could lead to a modulated ribosome that would in turn interpret the code in such a way as to express the same protein. As neither premise can reasonably be disputed, we must conclude that it is possible that an entirely undetectable transition in semantic closure could have occurred in biology.

The results we have presented demonstrate that there are multiple functionally distinct expressors that are capable of making a consistent interpretation of a given genome; consistent, in this context, means that the expressor's interpretation of the genome leads to its own expression. The expressor's interpretation of its own genetic material is a way of obtaining meaning through self-reference, in a way reminiscent of the formal systems discussed by Hofstadter in his seminal book *'Gödel, Escher, Bach'* [28]. Considering how these systems are related to each other, and if there is anything to be learned from determining if we have an analogy between systems or if they are actually isomorphic, is an intriguing notion that should be formally explored.

The stringmol UCA can be used as a tool to investigate many concepts that exist in and around its biological analogue. The results examined in §5.4 demonstrate the mutation of a genome that is classically heritable in a way that is well understood in biology. However, there are also heritable changes in that experiment that are not visible in the *in silico* genometic record: the mutated genome has no alteration in the section that encodes the original copier (figure 9*a*, step 2), yet the original copier is displaced by a novel copier that arises as a result of a transition between two semantically closed states. The effects of mutations in the expressor have the potential to be pleiotropic, as we have demonstrated. Given that we know transitions between semantically closed states have occurred in biology [2], it raises the question: have there been heritable changes in biological organisms that are simply not observable in the genetic record?

## 7. Software and primary data

Stringmol software is available at https://github.com/franticspider/stringmol/. These experiments were run using v. 0.2.3.3.

The configuration files and results files are available as a gzipped tarball at http://stringmol.york.ac.uk/suppmat/jrsi_2017.tgz.
